# Quantitative analysis of dynamic ^18^F-FDG PET/CT for measurement of lung inflammation

**DOI:** 10.1186/s13550-017-0291-2

**Published:** 2017-05-25

**Authors:** Christopher Coello, Marie Fisk, Divya Mohan, Frederick J. Wilson, Andrew P. Brown, Michael I. Polkey, Ian Wilkinson, Ruth Tal-Singer, Philip S. Murphy, Joseph Cheriyan, Roger N. Gunn

**Affiliations:** 1 Imanova Ltd., Centre for Imaging Sciences, Hammersmith Hospital, London, UK; 2 Division of Brain Sciences, Department of Medicine, Imperial College London, London, UK; 3 Experimental Medicine and Immunotherapeutics (EMIT) Division, Department of Medicine, University of Cambridge, Cambridge, UK; 4 NIHR Respiratory Biomedical Research Unit at the Royal Brompton and Harefield NHS Foundation Trust and Imperial College, London, UK; 5 GSK R&D, Cambridge, UK; 6 GSK R&D, Stevenage, UK; 7 Cambridge Clinical Trials Unit, Addenbrooke’s Hospital, Cambridge, UK; 8 GSK R&D, King of Prussia, PA USA; 9 Cambridge University Hospitals NHS Foundation Trust, University of Cambridge, Cambridge, UK; 10 Institute of Biomedical Engineering, Department of Engineering Science, University of Oxford, Oxford, UK

**Keywords:** PET, ^18^F-FDG, Lung inflammation, Modelling, COPD

## Abstract

**Background:**

An inflammatory reaction in the airways and lung parenchyma, comprised mainly of neutrophils and alveolar macrophages, is present in some patients with chronic obstructive pulmonary disease (COPD). Thoracic fluorodeoxyglucose (^18^F-FDG) positron emission tomography (PET) has been proposed as a promising imaging biomarker to assess this inflammation. We sought to introduce a fully quantitative analysis method and compare this with previously published studies based on the Patlak approach using a dataset comprising ^18^F-FDG PET scans from COPD subjects with elevated circulating inflammatory markers (fibrinogen) and matched healthy volunteers (HV). Dynamic ^18^F-FDG PET scans were obtained for high-fibrinogen (>2.8 g/l) COPD subjects (*N* = 10) and never smoking HV (*N* = 10). Lungs were segmented using co-registered computed tomography images and subregions (upper, middle and lower) were semi-automatically defined. A *q*uantitative analysis approach was developed, which corrects for the presence of *a*ir and *bl*ood in the *l*ung (*qABL* method), enabling direct estimation of the metabolic rate of FDG in lung tissue. A normalised Patlak analysis approach was also performed to enable comparison with previously published results. Effect sizes (Hedge’s g) were used to compare HV and COPD groups.

**Results:**

The qABL method detected no difference (Hedge’s g = 0.15 [−0.76 1.04]) in the tissue metabolic rate of FDG in the whole lung between HV (*μ* = 6.0 ± 1.9 × 10^−3^ ml cm^−3^ min^−1^) and COPD (*μ* = 5.7 ± 1.7 × 10^−3^ ml cm^−3^ min^−1^). However, analysis with the normalised Patlak approach detected a significant difference (Hedge’s g = −1.59 [−2.57 −0.48]) in whole lung between HV (*μ* = 2.9 ± 0.5 × 10^−3^ ml cm^−3^ min^−1^) and COPD (*μ* = 3.9 ± 0.7 × 10^−3^ ml cm^−3^ min^−1^). The normalised Patlak endpoint was shown to be a composite measure influenced by air volume, blood volume and actual uptake of ^18^F-FDG in lung tissue.

**Conclusions:**

We have introduced a quantitative analysis method that provides a direct estimate of the metabolic rate of FDG in lung tissue. This work provides further understanding of the underlying origin of the ^18^F-FDG signal in the lung in disease groups and helps interpreting changes following standard or novel therapies.

**Electronic supplementary material:**

The online version of this article (doi:10.1186/s13550-017-0291-2) contains supplementary material, which is available to authorized users.

## Background

Chronic obstructive pulmonary disease (COPD) is the third leading cause of death globally and is associated with significant morbidity and healthcare utilisation [1]. It is characterised in some patients by airflow limitation and persistent inflammation in the airways and lungs [2].

Thoracic^18^F-fluorodeoxyglucose (^18^F-FDG) positron emission tomography co-registered with computed tomography (PET/CT) has been proposed as a promising imaging modality [3] to investigate pathological processes, disease phenotype and pathophysiology and potentially evaluate novel therapies in COPD [4]. The metabolic rate constant derived from dynamic ^18^F-FDG tissue uptake in tissue has been shown to be a good marker of glycolytic activity in brain [5] and myocardial metabolism. An increased metabolic rate of ^18^F-FDG has previously been observed in COPD subjects vs controls [6–8], and this has been hypothesised to be a surrogate of pulmonary inflammation. In vitro assays of COPD lung tissue have shown that an increased metabolic activity is associated with primed and activated neutrophils [9], which are thought to drive inflammation in COPD patients.

Estimation of the ^18^F-FDG metabolic rate in lung tissue is the required parameter for assessing lung inflammation [10]. However, accurate measurement of the lung tissue metabolic rate is confounded by the presence of air and blood activity [11] in the region of interest (ROI). The presence of air in the lungs contributes to a reduction in the measured lung ROI activity as a consequence of partial volume effects [12, 13]. The presence of blood contributes a background radioactive signal to the lung ROI, artificially increasing the measured signal.

Estimation of the amount of air can be obtained using a co-registered attenuation correction CT (CT-AC) [12]. Methodology to correct the ^18^F-FDG lung uptake for air and blood contribution has been applied previously to patients with focal patterns of lung fibrosis [10]. In more diffuse diseases like COPD or asthma, quantification of ^18^F-FDG has been analysed using a Patlak graphical approach to estimate a metabolic activity outcome measure where the Patlak intercept is used as a surrogate to correct for air volume [14]. These previous studies [6–8] employing the Patlak method did not specifically include any correction for blood activity to the lung ROI. Additionally, the use of the Patlak intercept to correct for the amount of air in the lungs has not been validated.

In this work, we sought to introduce a *q*uantitative tracer kinetic analysis method that corrects for the presence of *a*ir and *b*lood in the *l*ung (*qABL* method), enabling a more accurate measurement of the lung tissue uptake of ^18^F-FDG. We compared this with the previously published normalised Patlak approach using a dataset comprising ^18^F-FDG PET scans from COPD subjects with elevated circulating inflammatory markers (fibrinogen), a recently qualified biomarker associated with exacerbations and mortality in COPD [15] and matched healthy never smokers (HV).

### Theory

The measured radioactivity concentration in a region of the lung can be expressed as the sum of three terms that reflect blood, tissue and air:

1 $$ {C}_M(t)={V}_B{C}_B(t)+\left(1-{V}_B-{V}_A\right){C}_T\left( t,{K}_1,{k}_2,{k}_3,{C}_B\right)+{V}_A{C}_A(t) $$

where *C*_*M*_(*t*) is the measured radioactivity concentration, *C*_*B*_(*t*) is the radioactivity concentration in blood, *C*_*T*_(*t*) is the radioactivity concentration in tissue, *C*_*A*_(*t*) is the radioactivity concentration in air, *V*_*A*_ is the fractional air volume in the ROI, *V*_*B*_ is the fractional blood volume in the ROI, and *K*_1_, *k*_2_, *k*_3_ are the compartmental model’s microparameters. For radiotracers administered intravenously such as ^18^F-FDG, the radioactivity concentration in air (*C*_*A*_(*t*)) can be considered negligible and so the third term of Eq. (1) is zero. The kinetics of ^18^F-FDG in tissue have been shown to be well described in humans by an irreversible two tissue compartmental model [16] with the metabolic rate constant (${K}_i=\frac{K_1{k}_3}{k_2+{k}_3}$) of ^18^F-FDG being the outcome measure of interest.

Using the measured activity in the ROI (*C*_*M*_(*t*)) and in blood (*C*_*B*_(*t*)), least square estimation of the five model parameters (*K*_1_, *k*_2_, *k*_3_, *V*_*A*_, *V*_*B*_) leads to problems of numerical unidentifiability. However, *V*_*A*_ can be estimated independently using CT imaging [12]. The proposed method uses this estimate of *V*_*A*_ (denoted ${V}_A^{CT}$) in Eq. (1). This leaves four parameters to be estimated from fitting to the dynamic PET data similar to the general model for dynamic brain PET data [17] with the exception that the tissue term is weighted by $\left(1-{V}_B-{V}_A^{CT}\right)$ instead of (1 − *V*_*B*_):

2 $$ {C}_M(t)={V}_B{C}_B(t)+\left(1-{V}_B-{V}_A^{\mathrm{CT}}\right){C}_T\left( t,{K}_1,{k}_2,{k}_3,{C}_B\right) $$

## Methods

The study is being conducted in accordance with Good Clinical Practice standards. The participating sites received favourable opinion from the local ethics committee and approval from the Administration of Radioactive Substances Advisory Committee. All patients provide informed written consent before enrolment in accordance with the Declaration of Helsinki.

### Patients and healthy volunteers

Baseline dynamic ^18^F-FDG PET/CT scans obtained from the EVOLUTION (COPD, *N* = 10) and the EVOLVE (HV, *N* = 10) studies were used for this methodological assessment. The subset of ten subjects with COPD from EVOLUTION were chosen to be matched for age and gender with the ten HV used from EVOLVE. These COPD subjects were aged between 50 and 85 years, with a body mass index less than 35 kg/m^2^ and a plasma fibrinogen level greater than 2.8 g/l at screening. The same acquisition protocol was applied in all scans.

The EVOLUTION trial (Evaluation of losmapimod in COPD patients stratified by fibrinogen [18], ClinicalTrials.gov, NCT01541852) is a double-blinded placebo-controlled multicentre randomised controlled trial which incorporates ^18^F-FDG PET/CT imaging of the lungs, aorta and carotids at baseline and approximately 16 weeks following treatment with losmapimod (p38 mitogen-activated protein kinase inhibitor: (GW856553, GlaxoSmithKline, Brentford, UK)) or placebo.

The EVOLVE study (REC 13/EE/0165, UK CRN ID 1513) is a cross-sectional multicentre study designed to evaluate lung and vascular inflammation by imaging subjects with COPD secondary to cigarette smoke compared with subjects with COPD secondary to alpha-1 antitrypsin deficiency and to compare these with subjects with obstructive sleep apnoea, healthy smokers and healthy never smoking controls (HV).

### Image acquisition

Each subject underwent a low-dose CT scan and a 60-min dynamic ^18^F-FDG PET scan following a bolus injection of 237.3 ± 10.4 MBq (2.81 ± 0.54 MBq/kg) of radiotracer (full details of the image acquisition are provided in Additional file 1).

### Image processing

**Fig. 1:**
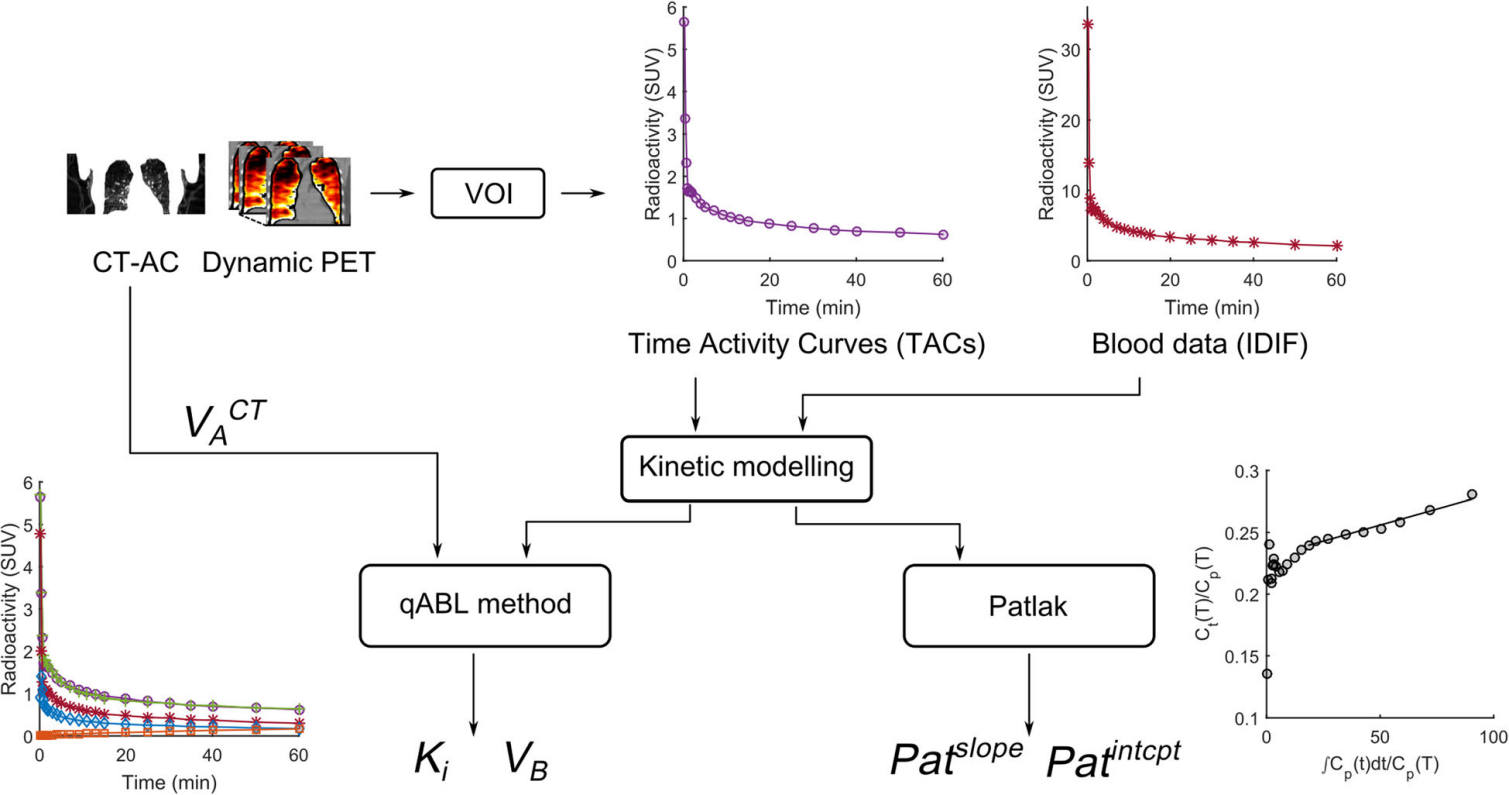
Overview of the kinetic modelling approaches implemented in this paper to analyse dynamic ^18^F-FDG lung PET data

The analysis pipeline (Fig. 1) was common to all PET-CT data. Except for the whole lung and pulmonary artery segmentation, the post-processing of the images and kinetic modelling was achieved using the Molecular Imaging and Kinetic Analysis Toolbox (MIAKAT™, www.miakat.org), a Matlab (The MathWorks Inc., Natick, MA, USA) toolbox. Description of the delineation of the whole lung (WL) mask can be found in the Additional file 1.

#### Regional time activity curves (TACs)

To study regional differences of ^18^F-FDG uptake, the downsampled WL mask was automatically subdivided into three regions of similar volume along the axial direction. The upper (UL), middle (ML) and lower (LL) lung masks were generated so that approximately each region equals one third of the total whole lung mask volume. Using the WL mask and the three subdivisions mentioned above, four time activity curves (TACs) were generated by calculating the mean regional activity for each time frame of the dynamic PET.

The standard uptake value (SUV) outcome measure was calculated for each TAC by dividing the average activity between 30 and 60 min by the injected dose per kg (kBq/kg).

#### CT-derived estimation of the fractional air volume

At the regional level, the fractional air volume in a given region R (WL, UL, ML and LL) was estimated [12]:

3 $$ {V}_A^{\mathrm{CT}}=1-\frac{\left({\overline{HU}}_R- H{U}_{\mathrm{air}}\right)}{\left( H{U}_{\mathrm{tissue}}- H{U}_{\mathrm{air}}\right)} $$

where ${V}_A^{CT}$ is the estimated fractional air volume, ${\overline{HU}}_R$ is the mean Hounsfield Unit (HU) in region R, *HU*_air_ is the HU of air (−1024), and *HU*_tissue_ is an approximation of the HU of lung tissue (40, taken from [12]). The air volume in a region can be computed by multiplying ${V}_A^{CT}$ by the volume of the region.

#### Estimation of the lung input function

An image-derived input function (IDIF) was estimated as follows: a volume of interest was defined in the descending aorta (DA). Guided by an averaged early frame (0–5 min) PET and the downsampled CT-AC, the centre of the DA was manually drawn on a slice-by-slice basis (axial slices) using a 1-cm (5 voxel)-diameter disk mask starting at the aorta arch. To minimise the partial volume effect, the ROI was drawn very centrally in the DA over a large range of axial slices. The aorta mask was then applied to the dynamic PET image, and the DA time activity curve (TAC) was extracted. The actual blood input function used, C_B_(t), included a correction for the plasma to whole blood ratio (1.056 ± 0.015) which was determined from venous samples collected after 5 min.

For each scan, a global time delay was estimated to account for the time separation between the radioactivity passing in the tissue of interest and the descending aorta: for delays spanning from −50 to 50 s, a one tissue compartmental model was fitted between the first 5 min of the delayed blood IF and the first 5 min of the WL TAC. The estimated delay was the delay generating the lowest residual sum of squares on the model fit.

### Kinetic modelling

**Fig. 2:**
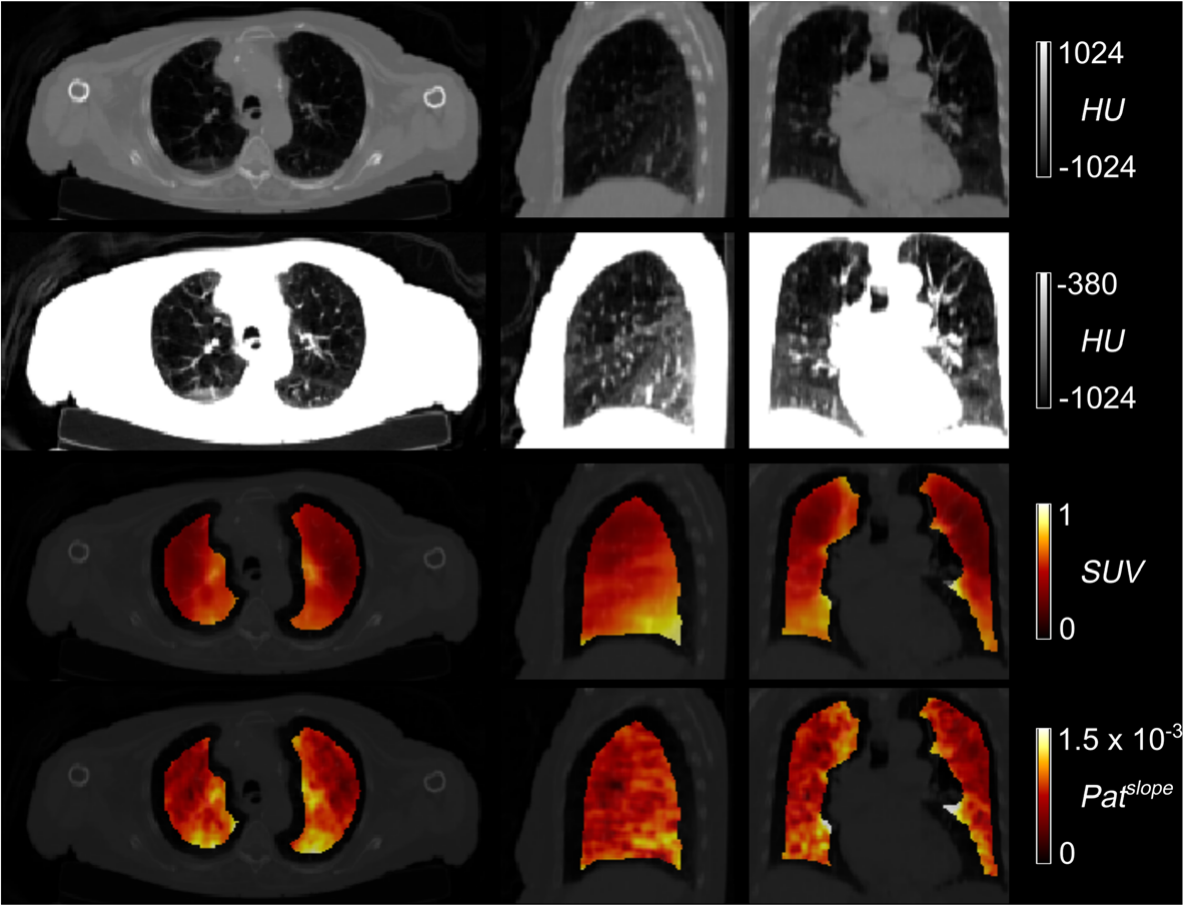
Axial (*left*), coronal (*middle*) and sagittal (*right*) slices for the CT-AC (first and second row) and the averaged (30–60 min) SUV (*third row*) and Pat^slope^ (fourth row) parametric images of a representative COPD patient (M, 66 years). The axial slice was chosen in the upper part of the lung as being representative of tissue loss in COPD. *CT-AC*: computed tomography attenuation correction, *SUV*: standardised uptake value

Kinetic modelling was performed combining Eq. (2) (that describes the tissue concentration of FDG within the ROI composed of air, blood and tissue) with the irreversible two tissue compartmental model describing the kinetics of FDG in the lung tissue itself. Combining these two levels of description is necessary to accurately estimate the metabolic rate of FDG in lung tissue only (Fig. 2). Because the method is a *q*uantitative analysis correcting for *a*ir and *b*lood in *l*ung tissue, the acronym *qABL* will be used to refer to this method.

The irreversible two tissue compartmental model (Fig. 2) is composed of a component with reversible kinetics characterised by the rate constant *K*_1_ and *k*_2_ and a component with irreversible kinetics (*k*_3_). The qABL method was implemented using a fitted fractional blood volume contribution leading to the estimation of four parameters (*K*_1_, *k*_2_, *k*_3_, *V*_*B*_) that were obtained by weighted least squares fitting. The metabolic rate constant of ^18^F-FDG corrected for air and blood, *K*_*i*_ (ml cm^−3^ min^−1^), was then calculated as

4 $$ {K}_i=\frac{K_1{k}_3}{k_2+{k}_3} $$

To enable comparison with previous studies [6–8], the Patlak graphical analysis [19] was also implemented with a linear start time (t*) fixed to 10 min. The slope (Pat^slope^) and the intercept (Pat^intcpt^) of the regression were extracted. The normalised metabolic rate $n{K}_i^{\mathrm{Pat}}$, used in previous studies [6–8] for quantifying lung tissue metabolism, was calculated as the ratio between the slope and intercept was calculated. As a consequence of the ROI containing air and blood (see Appendix 1), the outcome parameter $n{K}_i^{\mathrm{Pat}}$ is a composite measure that includes terms for the fractional air volume, fractional blood volume and the metabolic rate of ^18^F-FDG in lung tissue:

5 $$ n{K}_i^{\mathrm{Pat}}=\frac{{\mathrm{Pat}}^{\mathrm{slope}}}{{\mathrm{Pat}}^{\mathrm{intcpt}}}=\frac{\left(1-{V}_B-{V}_A\right){K}_i}{\left(1-{V}_B-{V}_A\right){V}_{\mathrm{ss}}+{V}_B} $$

with *K*_*i*_ being the tissue metabolic rate constant of 18F-FDG and *V*_ss_ the steady-state partition coefficient between tissue and plasma of non-phosphorylated FDG.

### Statistics

Differences between groups were assessed using the standardised corrected (or unbiased) effect size calculated using the Hedge’s g (abbreviated *g*) metric reported with the analytical 95% confidence interval (CI) measured using the Measures of Effect Size Toolbox [20]. Effect sizes are defined as small (*g* ≤ 0.2), medium (0.2 < *g* ≤ 0.8) and large (0.8 < *g*). Correlation was measured using Pearson’s correlation coefficient reported with the analytical 95% CI. A null hypothesis of zero effect between groups was rejected if the 95% CI did not contain the value 0. In this case, the group difference was named significant.

## Results

Demographics of the two cohorts are reported in Table 1.

**Table 1:** Demographics of the HV and the COPD cohorts

	HV	COPD
Age (years)	70 ± 7	71 ± 7
Gender (M/F)	09/01	08/02
Smokers (%)^a^	0	10
Mean total pack years (years)	0	42 ± 6
Body mass index (kg/m^2^)	26.4 ± 2.81	27.6 ± 4.20
FEV1 (l)	2.88 ± 0.64	1.39 ± 0.33
FEV1 (% predicted)	103 ± 15	48 ± 12

*FEV1* forced expiratory volume in 1 s

^a^% of current smokers (the rest being ex-smokers)

**Fig. 3:**
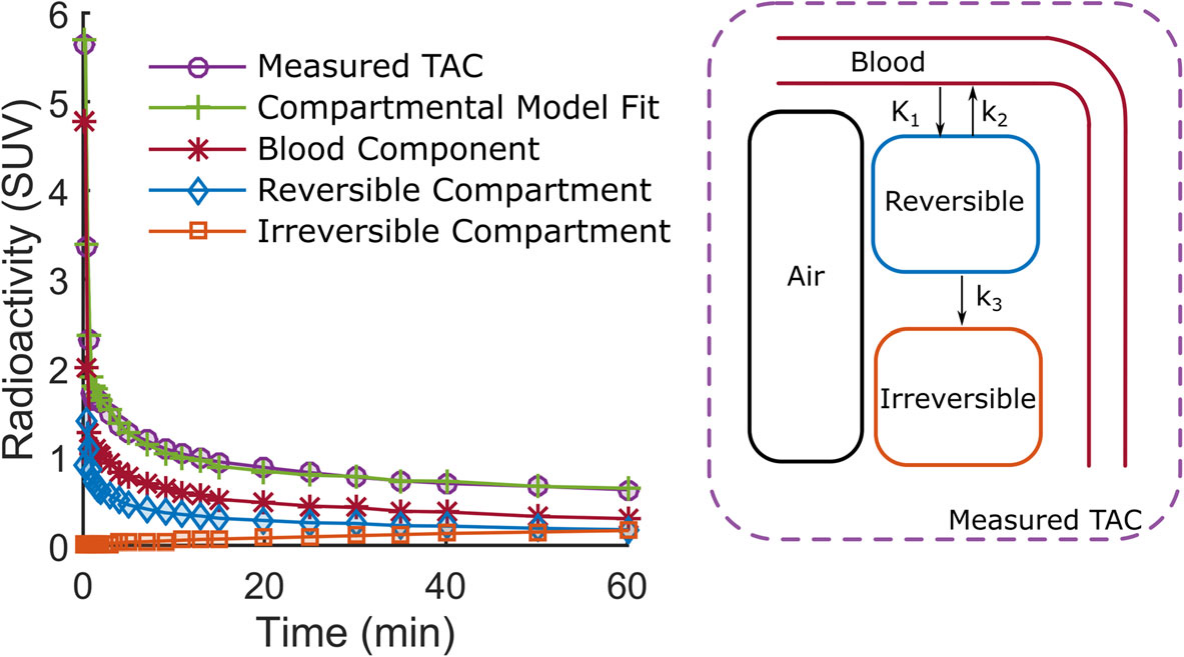
*Left*: representative TAC of a HV subject and estimated components obtained using the qABL method. *Right*: graphical representation of the different components involved in the qABL method: air, blood and tissue (tissue being modelled by an irreversible 2TC model for ^18^F-FDG)

The difference in density in the lungs observed on the CT (Fig. 2) is also observable on the SUV and Pat^slope^ images illustrating the need to account for lung density. Figure 3 shows a representative TAC of a HV subject and the model fit obtained using the qABL method. Different metrics derived from CT (${V}_A^{CT}$), static PET (SUV) and dynamic PET (Pat^slope^, Pat^intcpt^, $n{K}_i^{\mathrm{Pat}}$, *K*_*i*_, *V*_*B*_) images are reported in Table 2 for the whole lung and upper, middle and lower subregions. For each metric and region, the mean (*μ*) and coefficient of variation (CoV(%) = 100 × *σ*/*μ* where *σ* is the standard deviation of the metric) are reported.

**Table 2:** Parameter values across HV (*N* = 10) and COPD (*N* = 10) subjects in four lung ROIs

		Whole lung		Upper lung		Middle lung		Lower lung	
		HV	COPD	HV	COPD	HV	COPD	HV	COPD
*V*_*A*_^CT^	*μ*	0.72	0.79	0.74	0.80	0.72	0.79	0.68	0.78
	CoV (%)	6.0	5.2	5.0	7.3	6.0	5.1	7.7	4.4
SUV	*μ*	0.63	0.45	0.60	0.45	0.62	0.45	0.68	0.45
	CoV (%)	20	26	18	37	21	25	20	25
Pat^slope^	*μ* (×10^−3^)	0.61	0.51	0.54	0.52	0.57	0.51	0.73	0.50
	CoV (%)	21	22	30	35	26	22	31	33
Pat^intcpt^	*μ*	0.21	0.13	0.21	0.13	0.21	0.13	0.23	0.13
	CoV (%)	13	26	12	38	14	24	17	26
*nK*_*i*_^Pat^	*μ* (×10^−3^)	2.9	3.9	2.7	4.1	2.72	3.9	3.4	3.8
	CoV (%)	19	18	31	28	23	18	42	31
*K*_*i*_	*μ* (×10^−3^)	6.0	5.7	7.2	6.3	5.9	6.2	5.4	4.8
	CoV (%)	32	29	42	37	32	35	46	40
*V*_*B*_	*μ*	0.14	0.11	0.15	0.12	0.15	0.12	0.11	0.10
	CoV (%)	18	36	18	49	18	37	27	26

${V}_A^{CT}$ fractional air volume measured using CT-AC, *SUV* standardised uptake value, *Pat* ^*slope*^ Patlak slope, *Pat* ^*intcpt*^ Patlak intercept, $n{K}_i^{Pat}$ ratio Pat^slope^/Pat^intcpt^, *K*_*i*_ air and blood corrected metabolic rate of ^18^F-FDG in lung tissue, *V*_*B*_ fractional blood volume

### Metabolic rate of ^18^F-FDG in HV and COPD subjects

**Fig. 4:**
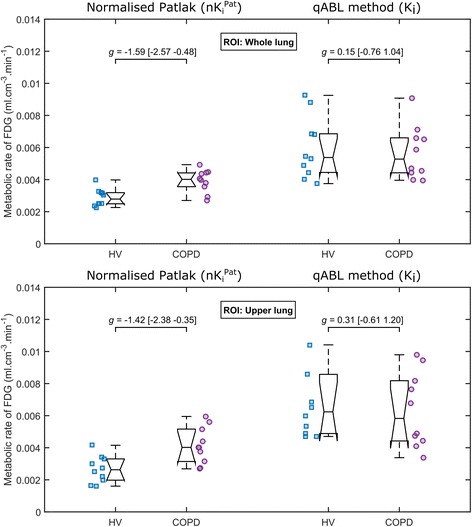
Metabolic rate of ^18^F-FDG in the whole lung (*top*) and the upper lung (*bottom*) estimated with normalised Patlak (*left*) and qABL method (*right*) in HV and COPD cohorts. Individual points are plotted together with median, *q*_1_ (first quartile) and *q*_3_ (third quartile). *Whiskers* extent: *q*_3_ − 1.5 * (*q*_3_ − *q*_1_) for the low boundary and *q*_3_ + 1.5 * (*q*_3_ − *q*_1_) for the high boundary

The metabolic rate of glucose estimated using the qABL method (*K*_*i*_) and the Patlak analysis ($n{K}_i^{\mathrm{Pat}}$) are displayed in Fig. 4 for each subject in the HV and COPD groups.

There was no significant difference in whole lung metabolic rate constant (*K*_*i*_) between the HV (*N* = 10, *μ* = 6.0 ± 1.9 × 10^−3^ ml cm^−3^ min^−1^) and COPD (*N* = 10, *μ* = 5.7 ± 1.7 × 10^−3^ ml cm^−3^ min^−1^) groups when estimated using the qABL method. The measured effect size was small in both the whole lung (*g* = 0.15 [−0.76 1.04]) and in the upper lung (UL: *g* = 0.31 [−0.61 1.20]).

In contrast, with the Patlak analysis, there was a significant difference in whole lung $n{K}_i^{\mathrm{Pat}}$ which was lower in the HV (*N* = 10, *μ* = 2.9 ± 0.5 × 10^−3^ ml cm^−3^ min^−1^) compared to COPD (*N* = 10, *μ* = 3.9 ± 0.7 × 10^−3^ ml cm^−3^ min^−1^) groups, with a large effect size (*g* = −1.59 [−2.57 −0.48]). A similarly large effect size was measured when the ROI was restricted to the upper lung (UL: *g* = −1.42 [−2.38 −0.35]).

### Fractional air and blood volume

**Fig. 5:**
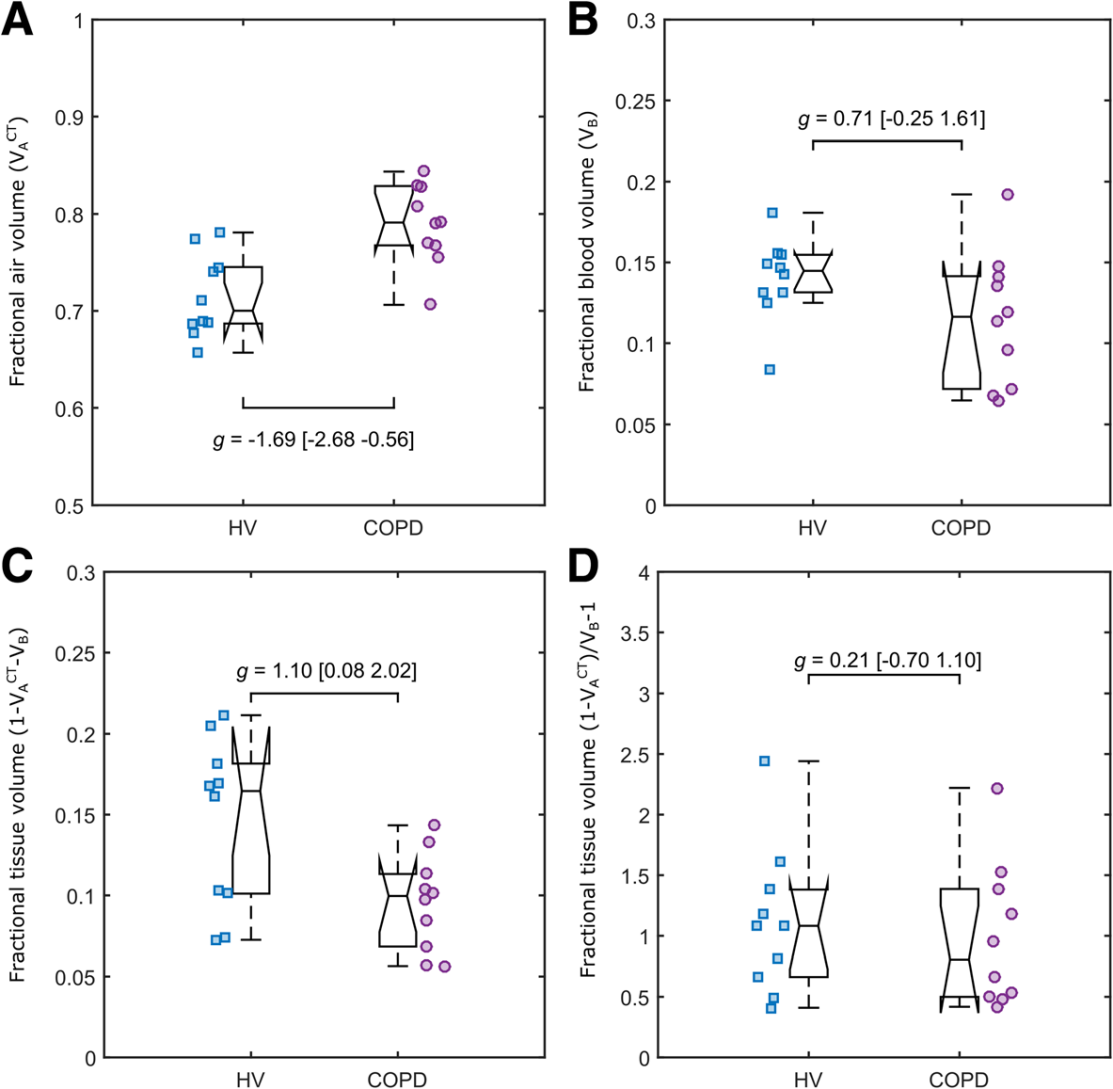
Group comparison of the fractional air volume ${V}_A^{CT}$ (**a**), fractional blood volume *V*_*B*_ (**b**), fractional tissue volume ($1-{V}_A^{CT}-{V}_B$) (**c**) and tissue to blood ratio (**d**) parameters in the whole lung obtained with the qABL method

The CT-derived fractional air volume (Table 2 and Fig. 5a) was, as expected, higher in COPD compared to HV (WL: *g* = −1.69 [−2.68 −0.56], UL: *g* = −1.15 [−2.08 −0.12]) consistent with the presence of emphysema in COPD. As a consequence, the fraction of the non-air (blood and tissue) component was smaller in COPD subjects than in HV. The fractional blood (*V*_*B*_) and the fractional tissue volume $\left(1-{V}_A^{CT}-{V}_B\right)$ were lower in COPD than in HV (Table 2, Fig. 5b, c), with a large effect size for both the WL (blood: *g* = 0.71 [−0.25 1.61], tissue: *g* = 1.10 [0.08 2.02]) and UL (blood: *g* = 0.71 [−0.25 1.61]). Medium effect size was measured for the fractional tissue volume in the UL (*g* = 0.63 [−0.32 1.53]). Finally, medium effect size (WL: *g* = −0.21 [−0.70 1.10], Fig. 5d) in the tissue to blood ratio ($\left(1-{V}_A^{CT}\right)/{V}_B-1$) was observed between COPD and HV with no significant difference between groups.

### Patlak intercept

**Fig. 6:**
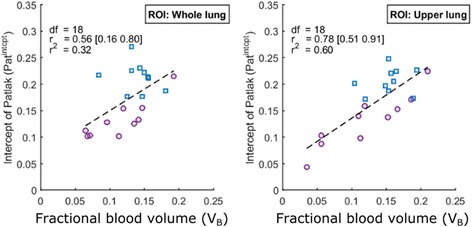
Scatter plot showing the relationship between the intercept of the graphical Patlak analysis (*Pat* ^*intcpt*^) and the fractional blood volume (*V*_*B*_). *Left* whole lung, *Right* upper lung, *Blue squares* HV, *violet circles* COPD

Because both the qABL and Patlak analysis were implemented on the same dataset, the relationship between the Patlak intercept and the fractional blood volume could be investigated. The scatter plot showing the relationship between the Patlak intercept (Pat^intcpt^) is shown in Fig. 6. The relationship between these variables is shown in Eq. A14 of Appendix 1. A significant correlation (WL: *r* = .56 [.16 .80], UL: *r* = .78 [.51 .91]) of the Patlak intercept to the fractional blood volume (*V*_*B*_) was measured.

## Discussion

The objective of this work was to introduce a quantitative method (noted qABL) for the measurement of the metabolic rate of ^18^F-FDG in lung tissue and to compare this with the previously published normalised Patlak approach using a dataset comprising ^18^F-FDG PET scans from COPD subjects with evidence of circulating inflammation (i.e. elevated circulating fibrinogen) and matched healthy volunteers.

The proposed qABL approach permits the direct estimation of the metabolic rate of ^18^F-FDG (*K*_*i*_) in lung tissue by appropriately correcting for the confounding contributions of air and blood. The inclusion of the estimation of the fractional air volume in the general Eq. (2) differs from a previously published method [10] by avoiding division of the parameters of interest (e.g. *K*_*i*_ or *V*_*T*_) by a correction factor that approaches 0. Thus, when the fraction of air increases, the method presented here will avoid artefactual increase of *K*_*i*_ in regions with emphysema. In addition, the model chosen for the tissue kinetics (in the term *C*_*T*_(*t*, *K*_1_, *k*_2_, *k*_3_, *C*_*B*_) of Eq. (2)) can be tailored to individual tracers enabling this methodology to be applied widely for quantitative PET lung imaging.

The proposed qABL method presented here shows no difference in the estimated metabolic rate of ^18^F-FDG (*K*_*i*_) between HV and high-fibrinogen COPD subjects in contrast to previously published studies containing a similar numbers of subjects using Patlak analysis [6–8]. Since inflammatory processes in the lung parenchyma in COPD have been widely described in the literature [21] and our measurements show no difference between healthy volunteers and COPD patients, we can infer that whole lung FDG may simply be insufficiently sensitive to detect the increased inflammation.

Applying the normalised Patlak approach ($n{K}_i^{\mathrm{Pat}}$) as used in three prior studies [6–8] to our dataset, we were able to replicate the magnitude of previously observed group differences between HV and high-fibrinogen COPD subjects (Table 3) with similar effect sizes.

**Table 3:** Comparison of *nK*_*i*_^Pat^ between our results and previously published

	ROI	HV		COPD		Group comparison		
		#	Mean ± Std	#	Mean ± Std	Abs diff.	% change	Effect size (g)
Ref [2]	WL	8	3.0 ± 1.2	10	4.2 ± 1.6	1.2	+40%	0.79
This dataset	WL	10	2.8 ± 0.4	10	3.9 ± 0.7	1.1	+39%	1.59
Ref [1]	UL	9	4.5 ± 0.9	10	6.1 ± 1.6	1.6	+36%	1.15
This dataset	UL	10	2.7 ± 0.8	10	4.1 ± 1.1	1.6	+64%	1.42

*WL* whole lung, *UL* upper lung

Nevertheless, in Appendix 1, we theoretically show that the normalised Patlak outcome measure includes terms for blood and air volume that introduce a bias. For example, in the absence of differences in *K*_*i*_, we show in Appendix 2 that changes in *V*_*B*_ and *V*_*A*_ observed in COPD lead to an artefactual ~30% increase in $n{K}_i^{\mathrm{Pat}}$. Thus, $n{K}_i^{\mathrm{Pat}}$ is not a direct measure of the lung tissue metabolic rate but is rather a composite outcome measure sensitive to changes in *K*_*i*_, *V*_*B*_ and *V*_*A*_, which makes it difficult to interpret. It may reflect disease severity and thus correlate with other disease severity biomarkers, but this correlation could be driven by any combination of differences in *K*_*i*_, *V*_*B*_ and/or *V*_*A*_.

The difference observed using normalised Patlak ($n{K}_i^{\mathrm{Pat}}$) might be driven by the other quantities *V*_*B*_ and/or *V*_*A*_ to which it is sensitive. When using normalised Patlak ($n{K}_i^{\mathrm{Pat}}$) as an estimate of the metabolic rate, the measured group difference between HV and COPD is driven by the denominator Pat^intcpt^ (Table 2, HV = 0.21 ± 0.03, COPD = 0.13 ± 0.05, *g* = 2.42 [1.11 3.53]) and not by the numerator Pat^slope^ (Table 2, HV = 0.61 ± 0.13, COPD = 0.51 ± 0.11, *g* = 0.64 [0.18 1.08]). Pat^intcpt^ is affected by changes in the air and blood volume in the ROI as shown in Eq. A13 of Appendix 1 and logically presents a high correlation to the fractional blood volume (*V*_*B*_) estimated with qABL method, giving further evidence that the group difference measured with normalised Patlak (*g* = −1.59 [−2.57 −0.48]) could be primarily due to the difference in blood volume between HV and COPD. Correction for blood using a fixed (not fitted) blood volume contribution is possible with graphical Patlak analysis, but this correction biases the outcome parameters as the blood volume varies between populations [22].

Some limitations of the two-tissue irreversible (2TC) model in lung tissues have been shown in preclinical models of acute lung injury [23, 24], and a model with an extra compartment (three-tissue irreversible (TEC) model) has been proposed to overcome these limitations. Schroeder et al. [23] proposed that lung kinetics could be classified as 2TC-type or TEC-type (Figs. 3 and 4 of Schroeder et al.). The kinetics observed in our study (Fig. 3) are of the 2TC-type, rather than the TEC-type, consistent with the exclusion of co-existing pathologies (i.e. acute lung injury) in our COPD population. If acute lung injury patients were to be analysed with the qABL method, a TEC model should be considered in place of the 2TC irreversible.

ROI mis-registration due to respiratory and cardiac cyclic movement [25] together with variability of the fractional air volume ${V}_A^{CT}$ due to the breathing cycle [26] have been identified as two potential limitations of the use of ${V}_A^{CT}$. The attenuation correction CT in this study was acquired during free breathing to achieve an average position of the lung. Higher air fraction in COPD (0.79) than in HV (0.72) measured with ${V}_A^{CT}$ is in agreement with previous findings [22]. In addition, the coefficient of variation of ${V}_A^{CT}$ (Table 2) in the whole lung for HV (6.0%) and COPD (5.2%) is low. These two elements give us confidence that ${V}_A^{CT}$ is a robust estimate for the fraction of air in the lungs.

The current investigation was limited to a sample size of 10 in each group, and so a change in the estimated *K*_*i*_ obtained with the qABL method cannot be ruled out in a larger study, although based on these data, if there is a signal, it is likely to be small.

A possible avenue for further work would be to investigate focal regions (anatomical lobes, individual voxels) where higher levels of inflammatory activity might be expected. For this purpose, either parametric images should be used or manually delineated regions focussing on specific pathology. Studying other populations with different levels of pulmonary inflammation would also give useful information and hence help us to understand whether ^18^F-FDG has more general utility for imaging lung inflammation. Correlation with systemic blood markers of inflammation (e.g. neutrophils, fibrinogen), with histology and with independent alternative measures of the quantities of interest, such as *V*_*B*_, would also be valuable. These results also suggest that development of other more specific tracers of the inflammatory process in COPD may be beneficial.

## Conclusions

We have introduced a quantitative method for the direct estimation of the metabolic rate of ^18^F-FDG PET in lung tissue that includes appropriate corrections for blood radioactivity and air. This work provides further understanding of the underlying origin of the ^18^F-FDG signal in the lung in disease groups and helps interpreting changes following standard or novel therapies.

## Figures and Tables

**Fig. 7:**
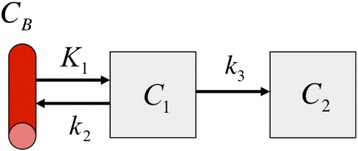
Irreversible 2TC model

## Appendix 1

In this appendix, the theoretical expression of the normalised Patlak metric is derived for regions containing air and blood.

The measure PET time activity from a region in the lung is given by,

A1 $$ {C}_M(t)={V}_B{C}_B(t)+\left(1-{V}_B-{V}_A\right){C}_T\left( t,{K}_1,{k}_2,{k}_3,{C}_B\right) $$

where C_B_ is the whole blood radioactivity concentration and C_T_ is the lung tissue radioactivity concentration (described by the irreversible two tissue compartment (2TC) model (Appendix Figure A1) with the micro parameters *K*_1_, *k*_2_ and *k*_3_).

The irreversible 2TC model is described by a set of differential equations,

A2 $$ \frac{d{ C}_1(t)}{ d t}={K}_1{C}_B(t)-{k}_2{C}_1(t)-{k}_3{C}_1(t) $$

A3 $$ \frac{d{ C}_2(t)}{ d t}={k}_3{C}_1(t) $$

These differential equations can be solved using the method of Laplace transform to derive the following analytical solutions,

A4 $$ {C}_1(t)={K}_1{e}^{-\left({k}_2+{k}_3\right) t}\otimes {C}_B(t) $$

A5 $$ {C}_2(t)=\frac{K_1{k}_3}{k_2+{k}_3}\left(1-{e}^{-\left({k}_2+{k}_3\right) t}\right)\otimes {C}_B(t) $$

where ⊗ is the convolution operator. The total tissue signal C_T_(t) (=C_1_(t)+C_2_(t)) is given by,

A6 $$ {C}_T(t)=\left(\frac{K_1{k}_2}{k_2+{k}_3}{e}^{-\left({k}_2+{k}_3\right) t}+\frac{K_1{k}_3}{k_2+{k}_3}\right)\otimes {C}_B(t) $$

The standard Patlak equation consistent with the irreversible 2TC ^18^F-FDG model can be derived by rearranging equation (A6) as,

A7 $$ {C}_T(t)={K}_i{\displaystyle {\int}_0^t{C}_B\left(\tau \right) d\tau +\frac{k_2}{k_2+{k}_3}{C}_1(t)} $$

where

$$ {K}_i=\frac{K_1{k}_3}{k_2+{k}_3} $$

Substituting (A7) into (A1) and dividing by C_B_(t) yields,

A8 $$ \frac{C_M(t)}{C_B(t)}={V}_B+\left(1-{V}_B-{V}_A\right)\left[{K}_i\frac{{\displaystyle {\int}_0^t{C}_B\left(\tau \right) d\tau}}{C_B(t)}+\frac{k_2}{k_2+{k}_3}\frac{C_1(t)}{C_B(t)}\right] $$

The Patlak method assumes that the free ^18^F-FDG in tissue has reached its steady state after a time *t**, such that,

A9 $$ {\left.\frac{C_1(t)}{C_B(t)}\right|}^{t>{t}^{*}}=\frac{K_1}{k_2+{k}_3} $$

Substituting (A9) into (A8) yields,

A10 $$ \frac{C_M(t)}{C_B(t)}={V}_B+\left(1-{V}_B-{V}_A\right)\left[{K}_i\frac{{\displaystyle {\int}_0^t{C}_B\left(\tau \right) d\tau}}{C_B(t)}+\frac{K_1{k}_2}{{\left({k}_2+{k}_3\right)}^2}\right] $$

which simplifies to,

A11 $$ \frac{C_M(t)}{C_B(t)}=\left(1-{V}_B-{V}_A\right){V}_{ss}+{V}_B+\left(1-{V}_B-{V}_A\right){K}_i\frac{{\displaystyle {\int}_0^t{C}_B\left(\tau \right) d\tau}}{C_B(t)} $$

where

$$ {V}_{ss}=\frac{K_1{k}_2}{{\left({k}_2+{k}_3\right)}^2} $$

Thus, when applying the Patlak analysis method of the form,

A12 $$ \frac{C_M(t)}{C_B(t)}= Pa{t}^{slope}\frac{{\displaystyle {\int}_0^t{C}_B\left(\tau \right) d\tau}}{C_B(t)}+ Pa{t}^{intcpt} $$

to lung data, it can be shown (by equating (A11) and (A12)) that the actual measured slope and intercept are:

A13 $$ Pa{t}^{slope}=\left(1-{V}_B-{V}_A\right){K}_i $$

A14 $$ Pa{t}^{intcpt}=\left(1-{V}_B-{V}_A\right){V}_{ss}+{V}_B $$

Thus the normalized Patlak outcome measure $\left( n{K}_i^{Pa t}=\frac{Pa{ t}^{slope}}{Pa{ t}^{i ntcpt}}\right)$ can be shown to be a composite parameter that includes terms for fractional blood volume (*V*_*B*_), fractional air volume (*V*_*A*_) and the metabolic rate of glucose in lung tissue (*K*_*i*_).

A13 $$ n{K}_i^{Pa t}=\frac{Pa{ t}^{slope}}{Pa{ t}^{i ntcpt}}=\frac{\left(1-{V}_B-{V}_A\right){K}_i}{\left(1-{V}_B-{V}_A\right){V}_{ss}+{V}_B} $$

## Appendix 2

Application of Appendix 1: theoretical prediction of the group difference between HV and COPD groups when there is an absence of difference of metabolic rate of ^18^F-FDG in tissue i.e. K_i_(HV) = K_i_(COPD).

Fractional air volume (V_A_) and fractional blood volume (V_B_) estimates were obtained from previously published quantitative H_2_^15^O and C^15^O scans^25^:

- HV : V_A_ = 0.74, V_B_ = 0.16
- COPD : V_A_ = 0.85, V_B_ = 0.08

An estimate of K_i_ and V_ss_ from the healthy volunteers was derived from Table 2:

- K_i_ = 6.10 x 10^-3^ ml.cm-3.min-1
- V_ss_ = 0.50 ml.cm^-3^

These parameters were fixed for both HV and COPD with only V_A_ and V_B_ being altered between the groups.

For HV,

$$ n{K}_i^{Pat}(HV)=\frac{\left(1-{V}_B(HV)-{V}_A(HV)\right){K}_i}{\left(1-{V}_B(HV)-{V}_A(HV)\right){V}_{ss}+{V}_B(HV)}=2.9 \times {10}^{-3} $$

For COPD,

$$ n{K}_i^{Pat}(COPD)=\frac{\left(1-{V}_B(COPD)-{V}_A(COPD)\right){K}_i}{\left(1-{V}_B(COPD)-{V}_A(COPD)\right){V}_{ss}+{V}_B(COPD)}=3.8 \times {10}^{-3} $$

Theory predicts a 31% increase in $n{K}_i^{Pat}$ for COPD compared to HV in the absence of differences in the metabolic rate of ^18^F-FDG in lung tissue.

## Additional file

Additional file 1: Supplemental data for quantitative analysis of dynamic ^18^F-FDG PET/CT for measurement of lung inflammation. (DOCX 318 kb)
