# Supplementary material for: Quantitative analysis of dynamic 18F-FDG PET/CT for measurement of lung inflammation
Source: EJNMMI Res. 2017 May 25;7:47. doi: 10.1186/s13550-017-0291-2 (PMC5445063; doi:10.1186/s13550-017-0291-2)
Supplement: Additional file 1: — Supplemental data for quantitative analysis of dynamic 18F-FDG PET/CT for measurement of lung inflammation. (DOCX 318 kb) [file 13550_2017_291_MOESM1_ESM.docx]

Supplemental Data for Quantitative analysis of dynamic ^18^F-FDG PET/CT for measurement of lung inflammation

# Methods

## COPD Disease Severity: spirometry measures

Spirometry was performed post bronchodilator and the best of three manoeuvres was accepted (CareFusion, San Diego, USA or using in-hospital body plethysmography) according to American Thoracic Society guidelines. Forced Expiratory Volume in 1 second (FEV1), Forced Vital Capacity (FVC), percentage predicted FEV1 (FEV1%) and FVC (FVC%) values for each participant were recorded.

## Image acquisition protocol

All subjects (N=10 with COPD and N=10 healthy volunteers) were scanned either at Imanova Ltd (London) on a Siemens Biograph TruePoint 6 PET-CT scanner (N=1 COPD and N=1 HV) or at the Addenbrooke's PET-CT Unit (Cambridge) on a GE Discovery PET-CT scanner (N=9 COPD and N=9 HV). After 6 hours of fasting, blood glucose level were measured and subjects were included if glucose concentration was <11mmol/L. The imaging procedure included an initial attenuation correction CT acquired with subject breathing freely, followed by ^18^F-FDG administered intravenously and finally a 60 minute dynamic PET scan. Injected dose range from 220.7 to 256.4 MBq (mean: 237.3 MBq, std: 10.4 MBq). During the scan, 12 discrete venous blood samples of approximately 5ml were drawn through an intravenous cannula inserted into the antecubital vein to measure whole blood and plasma radioactivity (sample times: 1, 2, 3, 4, 5, 7, 10, 15, 20, 30, 45 and 60 minutes).

List-mode data were binned into 23 frames (8x15s, 3x60s, 5x120s, 5x300s, 2x600s) and histograms were reconstructed at either 2mm nominal slice thickness using DIFT (Siemens) or 3.27mm nominal slice thickness using 3D Fore FBP (GE). Images were corrected for decay, attenuation, scatter and dead time. Finally, multiple 2D DICOM files were converted to a single 4D volume file in Nifti format. Dynamic PET data consisted of 23 volumes of 256 x 256 x 111 voxels (Siemens) or 256 x 256 x 47 voxels (GE) with a voxel size of 2.056 x 2.056 x 2 mm^3^ (Siemens) or 2.73 x 2.73 x 3.27 mm^3^ (GE).

Attenuation correction CT (CT-AC) was used for attenuation correction and for subsequent definition of volumes of interest. Therefore, multiple 2D DICOM files were converted to a single 3D volume file in Nifti format. CT data consisted of 512 x 512 x 111 voxels (Siemens) or 512 x 512 x 47 voxels (GE) with a voxel size of 1.37 x 1.37 x 2 mm^3^ (Siemens) or 1.37 x 1.37 x 3.27 mm^3^ (GE).

## Image Processing

Using Analyze 11.0 (AnalyzeDirect, Inc., Overland Park, KS), the CT-AC images were segmented using a semi-automatic intensity-based segmentation tool. The low threshold was chosen to be -1022 to remove airways and the high threshold -380 to avoid inclusion of big arteries^1^. The whole lung (WL) mask was then manually modified to remove any obvious airway inclusion. On a slice-by-slice manner, starting at the apex, the operator followed the trachea to the interface between primary bronchi and lung parenchyma: any voxel corresponding to the airways initially included in the WL mask was then excluded.

Dynamic PET-CT volumes and associated WL mask were resliced with isotropic voxel size (2 x 2 x 2 mm^3^ for PET and 1.37 x 1.37 x 1.37 mm^3^ for CT) and the CT-AC and WL mask were downsampled to match the PET voxel size (2 x 2 x 2 mm^3^).

Subsequently, a series of morphological operations were applied to the WL mask. First, a closing operation of the WL mask by a five voxel (1cm) diameter disk was achieved to homogenize the mask where the presence of vessels could have led to "holes" in the mask. Second, an additional erosion operation of the resulting WL mask by a five voxel (1cm) diameter disk was achieved to remove abnormal uptake that could exist on the edge of the lungs due to respiratory and other types of motion during the PET acquisition. Finally, a visual assessment of the PET uptake at the interface between the diaphragm and the lungs was done and further manual erosion of the WL mask was performed at this interface if liver uptake was still spilling into the WL mask.

## Metabolic rate of ^18^F-FDG in HV and COPD subjects

### Relationship to spirometry scores

The scatter plot showing the relationship between the metabolic rate constant of ^18^F-FDG ($K_{i}$) and a clinical spirometry measure (FEV1 percentage predicted or FEV1%) is presented in Figure S2. The correlation between the two measures is very weak, regardless of the ROI investigated (WL: *r* = .28 [-.43 .77], *r^2^* = .02, UL: r = .41 [-.30 .83], *r^2^* = .04). No correlation between the metabolic rate of ^18^F-FDG and spirometry scores (FEV1%) was observed (Figure S2), contrary to previous publications^8^ based on normalised Patlak (${nK}_{i}^{Pat}$).

Figure S2. Evolution of the metabolic rate of ^18^F-FDG (K_i_) in the whole lung (left) and the upper lung (right) with the FEV1 (% predicted)


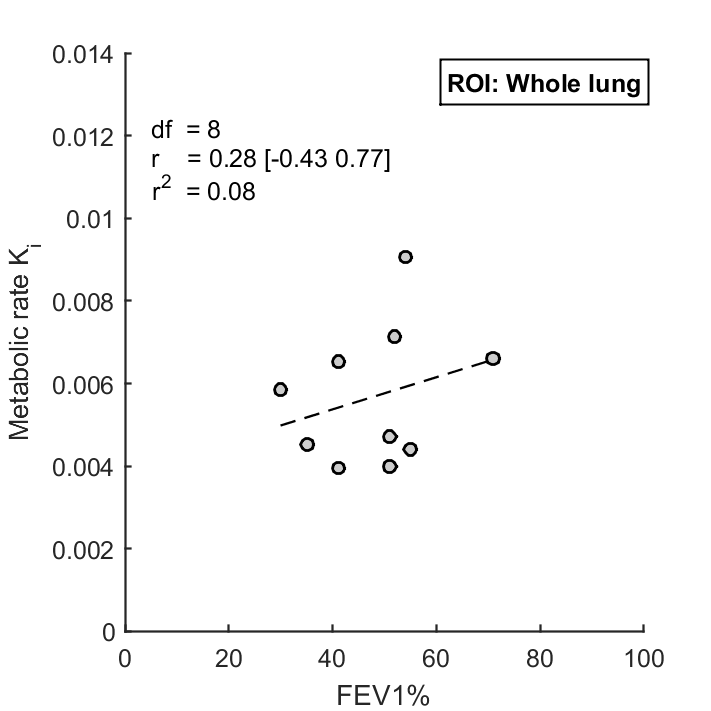

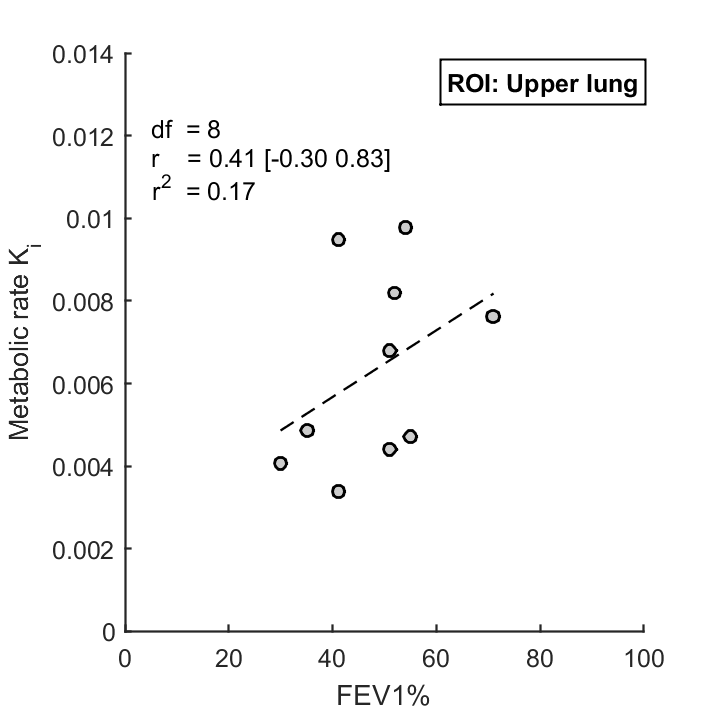


## Air volume, blood volume and Patlak intercept

### Relationship to spirometry scores

The scatter plot showing the relationship between the fraction of the three components of the lung (air, blood and tissue) and FEV1 percentage predicted (FEV1%) is reported in Figure S3 for the whole lung.

Figure S3. Scatter plot showing the relationship between the air volume (A), blood volume (B), tissue volume (C) and tissue to blood ratio (D) parameters and the FEV1% in the whole lung

| 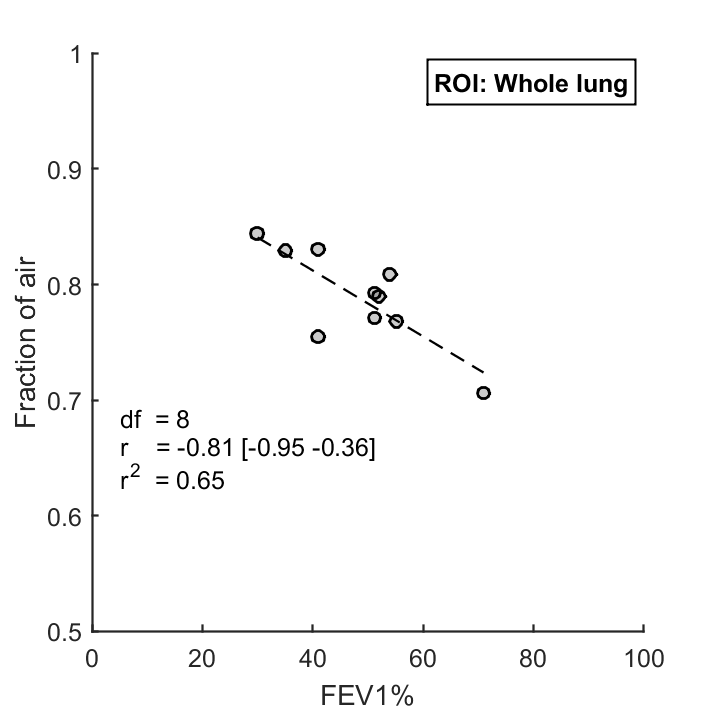A | 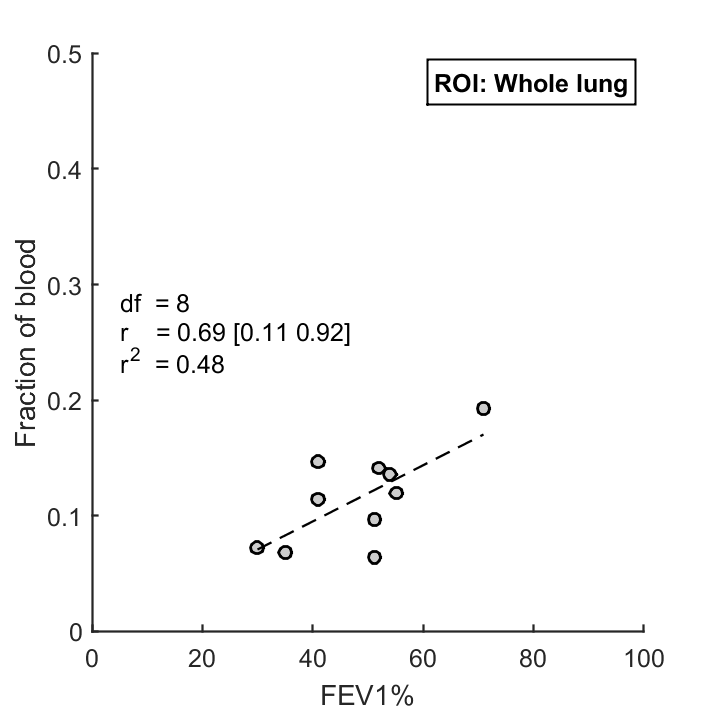B |
| --- | --- |
| 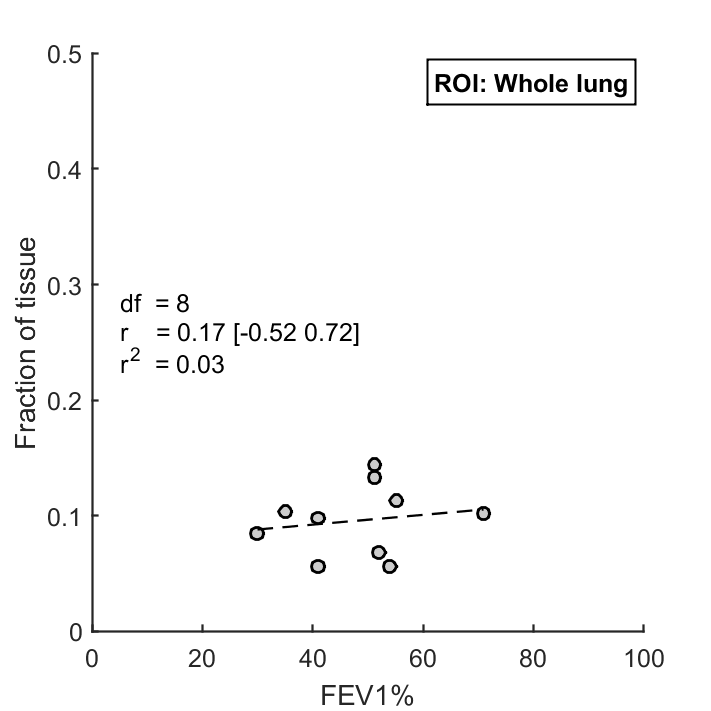C | 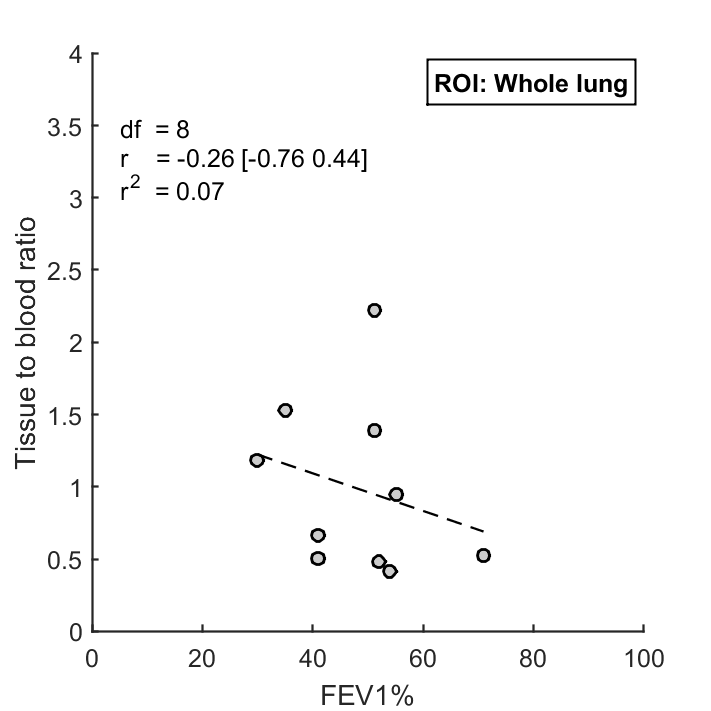D |

The fraction of air in the whole lung derived from the CT-AC ($V_{A}^{CT}$) shows a large negative (*r* = -.81 [-.95 -.36], *r^2^*=.65) correlation with FEV1% (Figure S3A). The fraction of blood in the whole lung ($V_{B}$) shows a large positive (*r* = .69 [.11 .92], *r^2^*=.48) correlation with FEV1% (Figure S3B) whereas the fraction of tissue is not correlated with FEV1% (*r* = .17 [-.52 .72], *r^2^*=.03) (Figure S3C). Finally, the ratio between the fraction of tissue and the fraction of blood ( ${{(1-V}_{A}^{CT})}/{V_{B}}-1$ ) in the whole lung is reported on the right of (Figure S3D). The ratio of tissue to blood is very weekly negatively correlated (*r* = -.26 [-.76 .44], *r^2^* = .07) with FEV1%.

# Discussion

The negative correlation between upper lung normalised Patlak and FEV1% (^8^, r(10)=-.85) was replicated, albeit with a lower level of correlation (Supplemental Figure S4, right, r(10)=-.42 [-.83 .28], r^2^ = .18).

Figure S4. Scatter plot showing the relationship between the normalised Patlak (nK_i_^Pat^) estimate of the metabolic rate of ^18^F-FDG in the whole lung (left) and the upper lung (right) and the FEV1 (% predicted) score.


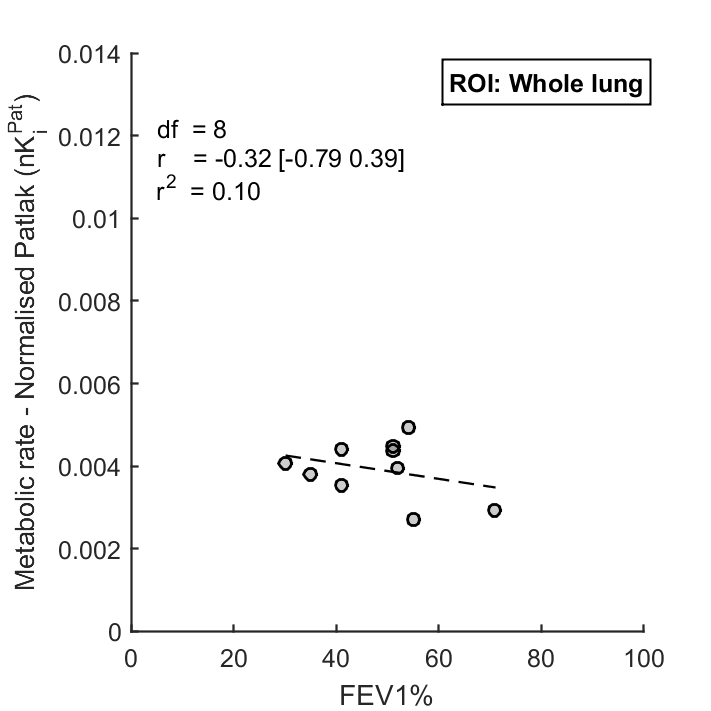

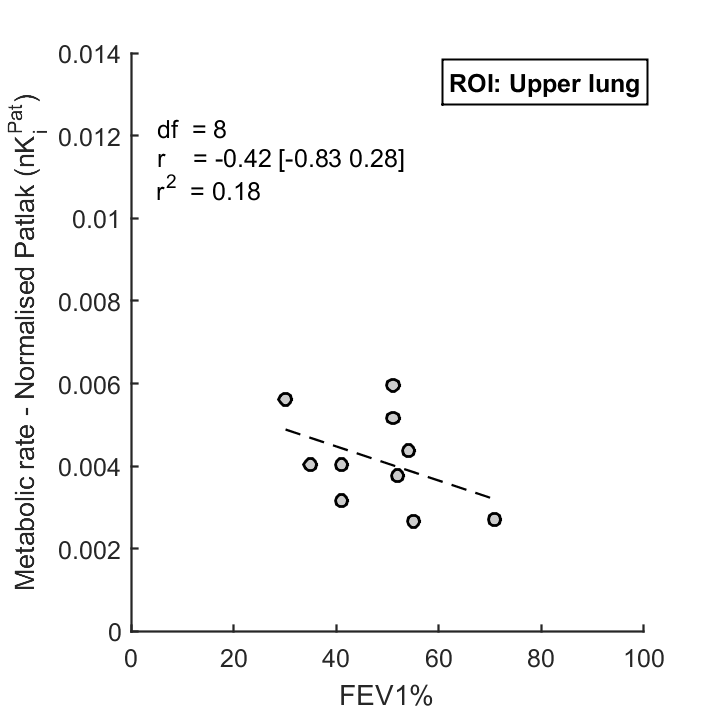


# Appendix S1

Application of Appendix 1: theoretical prediction of the group difference between HV and COPD groups when there is an absence of difference of metabolic rate of ^18^F-FDG in tissue ($K_{i}\left( HV \right)= K_{i}(COPD)$)

Air volume (V_A_) and blood volume (V_B_) estimates were obtained from previously published quantitative H_2_^15^O and C^15^O scans^3^:

HV : V_A_ = 0.74, V_B_ = 0.16

COPD : V_A_ = 0.85, V_B_ = 0.08

An estimate of K_i_ and V_ss_ from the healthy volunteers was derived from Table 2:

K_i_ = 6.10 x 10^-3^ ml.cm^-3^.min^-1^, V_ss_ = 0.50 ml.cm^-3^. These parameters were fixed for both HV and COPD with only V_A_ and V_B_ being altered between the groups.

For HV,

$$nK_{i}^{Pat}\left( HV \right)=\frac{\left( 1-V_{B}\left( HV \right)-V_{A}\left( HV \right) \right)K_{i}}{{\left( 1-V_{B}\left( HV \right)-V_{A}\left( HV \right) \right)V}_{ss}+V_{B}\left( HV \right)}=2.9 \times{10}^{-3}$$

For COPD,

$$nK_{i}^{Pat}\left( COPD \right)=\frac{\left( 1-V_{B}\left( COPD \right)-V_{A}\left( COPD \right) \right)K_{i}}{{\left( 1-V_{B}\left( COPD \right)-V_{A}\left( COPD \right) \right)V}_{ss}+V_{B}\left( COPD \right)}=3.8 \times{10}^{-3}$$

Theory predicts a 31 % increase in $nK_{i}^{Pat}$for COPD vs HV in the absence of differences in the metabolic rate of glucose in lung tissue.

# REFERENCES

1. Subramanian DR, Jenkins L, Edgar R, Quraishi N, Stockley R a., Parr DG. Assessment of pulmonary neutrophilic inflammation in emphysema by quantitative positron emission tomography. *Am J Respir Crit Care Med*. 2012;186(11):1125-1132. doi:10.1164/rccm.201201-0051OC.

2. Chen DL, Azulay D-O, Atkinson JJ, et al. Reproducibility Of Positron Emission Tomography (PET)-Measured [18F]Fluorodeoxyglucose ([18F]FDG) Uptake As A Marker Of Lung Inflammation In Chronic Obstructive Pulmonary Disease (COPD). In: Society AT, ed. *American Thoracic Society International Conference Abstracts*. Vol American Thoracic Society; 2011:A6449-A6449. doi:10.1164/ajrccm-conference.2011.183.1_MeetingAbstracts.A6449.

3. Rhodes CG, Hughes JMB. Pulmonary studies using positron emission tomography. *Eur Respir J*. 1995;8(6):1001-1017. doi:10.1183/09031936.95.08061001.
